# Effect of breastfeeding on metabolic-related outcomes in women with previous gestational diabetes mellitus: A protocol for systematic review and meta-analysis

**DOI:** 10.1097/MD.0000000000024716

**Published:** 2021-02-26

**Authors:** Bingfeng Zhou, Jun Zhou

**Affiliations:** a Department of Obstetrics, Affiliated Hospital of Chengde Medical University, Chengde, Hebei; b Department of Obstetrics, Shenzhen People's Hospital, Shenzhen, China.

**Keywords:** breastfeeding, diabetes, gestational diabetes mellitus, metabolic-related outcomes

## Abstract

**Background:**

This meta-analysis was to systematically investigate the effect of breastfeeding on metabolic-related outcomes in women with previous gestational diabetes mellitus (GDM).

**Methods:**

We will search the online databases of Relevant studies were searched in Pubmed, Embase, Cochrane Library, Web of Science. Relative risk and weighted mean difference with 95% confidence interval will pooled using Stata14.0 software.

**Conclusion:**

Our meta-analysis will explore the effect of breastfeeding on metabolic-related outcomes in women with previous GDM and may provide effective treatment options of GDM.

**OSF registration number:**

10.17605/OSF.IO/HA5U8

## 1 Introduction

Gestational diabetes mellitus (GDM) is a kind of most frequent metabolic complications, with the definition of glucose intolerance with first-episode or recognition during gestation.^[1]^ It is an important public health issue with the occurrence of 9.8% to 25.5%, and the incidence increases annually.^[2,3]^ Although most GDM women may return to normal glucose tolerance after delivery, the history of GDM brings long-term health risks later in their lives.^[4,5]^ Accumulating studies reveals that the GDM is correlated with the higher prevalence of diabetes.^[6,7]^ A systematic review of 20 studies demonstrated that GDM women had 7 times higher risk of type 2 diabetes mellitus (T2DM) development later in life than those without GDM.^[8]^ Therefore, women with a history of GDM are the main target group for diabetes prevention.

The positive effects of breastfeeding (BF) are mainly contributed to the health of children.^[9]^ Studies reported that mothers can also benefit from BF.^[10–12]^ BF is associated with the consumption of substantial energy, approximately 480 kcal/day during lactation.^[13]^ Thus, the modifiable postpartum behavior BF is generally considered to improve glucose and lipid metabolism and enhance insulin sensitivity, and the advantageous metabolic effects persist after weaning.^[14–16]^ Studies have found that the BF for more than 4 weeks can reduce the risk of T2DM development by 20% to 50% in women with previous GDM.^[17,18]^ However, some studies failed to show the positive effects of BF.^[19,20]^

Effects of BF on metabolic-related outcomes in women with prior GDM have not been well illustrated. In our meta-analysis, we will collect related articles from databases for investigation and comprehensive analysis to explore the metabolic impact of BF in women with prior GDM.

## 2 Methods

### 2.1 Protocol registration

Prospective registration of this study has been approved by the Open Science Framework (OSF) registries (https://osf.io/registries), (registration number: 10.17605/OSF.IO/HA5U8) For this type of article, there is no need of ethics approval and informed consent.

### 2.2 Search strategy

**Figure 1:**
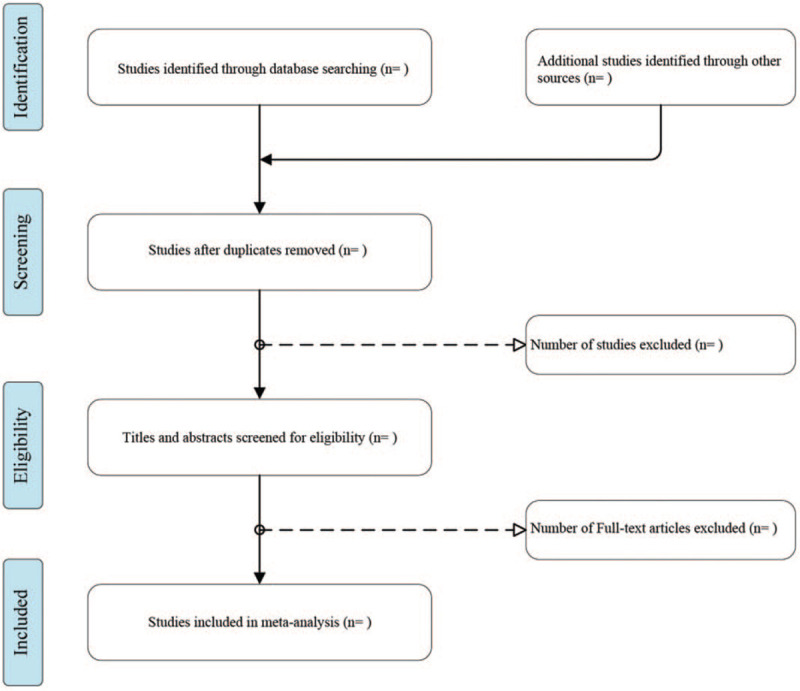
Flow diagram of inclusive and exclusive criteria.

Literature will be retrieved from Pubmed, Embase, Cochrane Library, Web of Science. The English search terms will be included ‘Breast Feeding [Mesh]’ OR ‘Feeding, Breast’ OR ‘Breastfeeding’ OR ‘Breast Feeding, Exclusive’ OR ‘Exclusive Breast Feeding’ OR ‘Breastfeeding, Exclusive’ OR ‘Exclusive Breastfeeding’ OR ‘Lactation [Mesh]’ OR ‘Milk Secretion’ OR ‘Milk Secretions’ OR ‘Lactation, Prolonged’ OR ‘Lactations, Prolonged’ OR ‘Prolonged Lactation’ OR ‘Prolonged Lactations’ AND ‘Diabetes, Gestational [Mesh]’ OR ‘Diabetes, Pregnancy-Induced’ OR ‘Diabetes, Pregnancy Induced’ OR ‘Pregnancy-Induced Diabetes’ OR ‘Gestational Diabetes’ OR Diabetes Mellitus, Gestational’ OR ‘Gestational Diabetes Mellitus’ OR GDM’ OR ‘Gestational Diabetic’ OR ‘Diabetic Pregnancy’ (Fig. 1).

### 2.3 Selection criteria

Cohort studies will be included in this meta-analysis. The criteria for inclusion will contain:

(1) subjects with GDM;
(2) BF group: GDM women with BF;
(3) non-BF group: GDM women without BF;
(4) studies on the relationship between BF and diabetes and or metabolic parameters;
(5) language in English;
(6) balanced and comparable baseline data.

Exclusion criteria will be included:

(1) wrong and uncorrectable statistical method;
(2) duplicated reports;
(3) literature with incomplete data, ambiguous outcome, and unpublished treatment results;
(4) reports, reviews, meta-analysis;
(5) defective research design.

### 2.4 Diagnostic criteria

Patients who met the World Health Organization or International Association of Diabetes and Pregnancy Study Group criteria will be diagnosed with GDM. World Health Organization diagnostic criteria: fasting plasma glucose (FPG) ≥7.0 mmol/L or 2-hour postprandial blood glucose ≥7.8 mmol/L. International Association of Diabetes and Pregnancy Study Group diagnostic criteria: FPG ≥5.1 mmol/L or 1-hour postprandial blood glucose ≥10.0 mmol/L, or postprandial 2H blood glucose ≥8.5mmol/L. Diagnostic criteria for diabetes: FPG ≥7.0 mmol/L; random blood glucose ≥11.1 mmol/L with typical symptoms of diabetes (polydipsia, polyuria, polyphagia and weight loss); oral glucose tolerance test 2-hour blood glucose ≥11.1 mmol/L, and the diagnosis would be confirmed with one of three points. Pre-diabetes mellitus diagnostic criteria: impaired FPG (FPG: 6.1–7.0mmol/L) and/or impaired glucose tolerance (2-hour postprandial blood glucose: 7.8–11.1mmol/L). The BF is defined as feeding babies with the maternal milk at discharge or after delivery.

### 2.5 Methodological quality appraisal and data extraction

The quality of included articles will be assessed by Newcastle-Ottawa scale.^[21]^ The total score will be 10, with scores <5 as low-quality and scores ≥5 as high-quality.

Extracted data will be included first author, publication year, country, number of included patients, duration of follow up, age, quality assessment score and outcomes. Outcomes included incidence of diabetes, FPG, 2-hour postprandial blood glucose, incidence of pre-diabetes mellitus, fasting insulin, homeostasis model assessment of insulin resistance, triglycerides, cholesterol, high-density lipoprotein (HDL) cholesterol, low-density lipoprotein cholesterol and insulin sensitivity index.

### 2.6 Statistical analysis

All studies will be analyzed using Stata14.0 software (Stata Corporation, College Station, TX, USA). Relative risk and weighted mean difference will be statistics for counting data and measurement data respectively, each effect size will be expressed by 95% confidence interval. Heterogeneity test will be carried out for each outcome, and the random effect model will be performed if the heterogeneity statistics *I*^2^ ≥50%, otherwise the fixed effect model will be used for analysis. Sensitivity analysis will be performed for all outcomes. Subgroup analysis and meta regression will be used for outcome indicator which with statistical significance (*P* < .05) and *I*^2^ ≥50%, to explore the sources of heterogeneity. Only meta regression will be performed for outcome indicator which without statistical significance (*P* > .05) and *I*^2^ ≥50%. *P* < .05 meant that the difference will be statistically significant.

## 3 Discussion

A meta-analysis conducted by Shujian Ma et al. confirmed the positive effects of BF in protection against the development of T2DM related outcomes in midlife of women with prior GDM.^[22]^ The benefits of BF on women with recent GDM was also reported by Kjos in 1993.^[23]^ Ziegler et al found the relationship between BF and the decreased diabetes risk among women with previous GDM after delivery.^[17]^

The insulin sensitivity index is closely related to diabetes. In the case of sufficient secretion, improving insulin sensitivity is a good way to treat diabetes. Increased insulin sensitivity can improve the condition and control the blood sugar level better, resulting in a lower likelihood of diabetes. Butte et al. found a lower fasting insulin level in BF women than non-BF women at 6 months after delivery, while no difference in the fasting glucose level was found between two groups.^[24]^ Others observed a trend for increased fasting insulin levels and homeostasis model assessment of insulin resistance in non-BF women compared with BF mothers.^[25]^ A hypothesis of insulin-independent mechanism has also been suggested for the explanation of metabolic effects of BF in women with previous GDM.^[24]^ The glucose values in plasma decrease due to the preferential transformation of glucose to the mammary glands. This occurs in all BF women and plays an active role in those with a high risk of metabolic disorders.

BF may also have effects on the lipoprotein profiles. Kjos et al reported the positive effects of BF on lipoprotein profiles.^[23]^ They found an increased level of HDL cholesterol in the lactating group while no difference in the triglyceride, HDL cholesterol and total cholesterol levels were reported. A higher HDL cholesterol level in BF women has been exhibited by Gunderson et al.^[25]^

Above all, whether BF affects metabolic-related outcomes in women with prior GDM remains a topic of debate. Therefore, we will perform a meta-analysis to explore the effect of BF on metabolic-related outcomes in women with GDM.

## Author contributions

Bingfeng Zhou: Conceptualization, manuscript writing and editing, data collection and analysis; Jun Zhou: Conceptualization, supervision and revised the manuscript. All authors read and approved the final manuscript.

**Conceptualization:** Bingfeng Zhou, Jun Zhou.

**Data curation:** Bingfeng Zhou, Jun Zhou.

**Formal analysis:** Bingfeng Zhou, Jun Zhou.

**Writing – original draft:** Bingfeng Zhou, Jun Zhou.

**Writing – review & editing:** Bingfeng Zhou, Jun Zhou.
